# Large autologous ilium with periosteum for tibiotalar joint reconstruction in Rüedi-Allgöwer III or AO/OTA type C3 pilon fractures: a pilot study

**DOI:** 10.1186/s12891-020-03659-7

**Published:** 2020-09-25

**Authors:** Dong Li, Jiao Jiao Li, Yuanyuan Zhu, Fushan Hou, Yuan Li, Bin Zhao, Bin Wang

**Affiliations:** 1 Department of Orthopedic, Second Hospital of Shanxi Medical University, Taiyuan, China; 2 School of Biomedical Engineering, Faculty of Engineering and IT, University of Technology Sydney, Ultimo, NSW Australia; 3 Department of Pharmacy, Shanxi Medical University Second Affiliated Hospital, Taiyuan, China

**Keywords:** Tibial pilon fracture, Ilium, Periosteum, Open reduction and internal fixation (ORIF), Articular reconstruction

## Abstract

**Background:**

Management of Rüedi-Allgöwer III or AO/OTA type C3 pilon fracture presents numerous challenges to the orthopaedic surgeon. A joint preservation technique using a large autologous ilium with periosteum in combination with internal implant fixation was reported to improve the outcome of reconstruction**.**

**Methods:**

Twenty-five patients according to Tscherne/Oestern FxCO-I closed fracture and FxOI open fractures classification after Rüedi-Allgöwer III or AO/OTA type C3 pilon fracture received a large autologous ilium with periosteum for tibiotalar joint reconstruction and open reduction and internal fixation (ORIF), between March 2015 and September 2018. The visual analog scale (VAS), American Orthopaedic Foot & Ankle Society (AOFAS) score, and Burwell and Charnley criteria were used for outcome analysis.

**Results:**

Twenty patients with an average age of 45.2 years were followed for an average of 18.3 months. The VAS and AOFAS scores, and Burwell and Charnley ratings were recorded at the last follow-up after reconstructive surgery. Two patients developed redness and swelling at the wound site, but recovered after local care and dressing changes. No patient displayed deep surgical site infection, donor site complication, non-union or local complication during the final follow-up. The average bone union time was 18.3 months (range 3–36).

**Conclusions:**

Large autologous ilium with periosteum in combination with ORIF can be performed for tibiotalar joint reconstruction. This experimental procedure reduces the risk of post-operative complications following articular reconstruction for Rüedi-Allgöwer III or AO/OTA type C3 pilon fractures in short follow-up.

**Level of evidence:**

Level III, retrospective cohort study.

## Background

Unlike common ankle fractures, pilon fractures resulting from high-energy trauma are much more complex [1], and account for approximately 5 to 7% of all tibial fractures [2]. Recent data has demonstrated that the majority of patients are unable to regain their pre-operative work status, and experience greater reduction in post-operative quality of life compared to other orthopaedic procedures [3]. Furthermore, pilon fractures are associated with a high risk of would complications, and predispose the patient to developing post-traumatic osteoarthritis of the tibiotalar joint. The management of these fractures present numerous challenges to the orthopaedic surgeon, particularly for the Rüedi-Allgöwer III or AO/OTA type C3 pilon fracture, which represents severely comminuted fractures with impaction of the distal tibia and are heterogeneous in both injury pattern and treatment [4, 5].

Various methods for the pilon fracture reconstruction have been reported, including minimally invasive plate osteosynthesis (MIPO) [6], external fixation [7], open reduction and internal fixation (ORIF) [8], and intramedullary interlocking nails [9]. For cases combined with a risk of soft tissue and osseous sepsis, reconstructive techniques included early fixation, upgrading, primary arthrodesis, staged sequential posterior and anterior fixation, acute shortening, and trans-syndesmotic fibular plating [10]. Although the risk of infection and soft tissue complications may be reduced with the use of external fixation, this method may not allow optimal reduction of the joint surface and therefore may jeopardise long-term outcomes. Classic studies have reported the use of bone grafts in treating the bony defect after fracture reduction, but this has been associated with poor prognosis [11]. Consequently, there is a paucity of studies reporting the use of a large autologous ilium with periosteum for tibiotalar joint reconstruction in pilon fractures, and long-term outcomes following such treatment.

This study reports a single-stage surgical procedure for joint reconstruction in a cohort of patients with Rüedi-Allgöwer III or AO/OTA type C3 pilon fracture. The short-term outcomes of tibiotalar joint reconstruction are evaluated following the implantation of a large autologous ilium having periosteum together with ORIF. This procedure is associated with positive post-operative outcomes, and may help reduce the risk of complications, delay joint fusion or articular arthroplasty for post-traumatic ankle osteoarthritis.

## Methods

### Patient characteristics

**Fig. 1:**
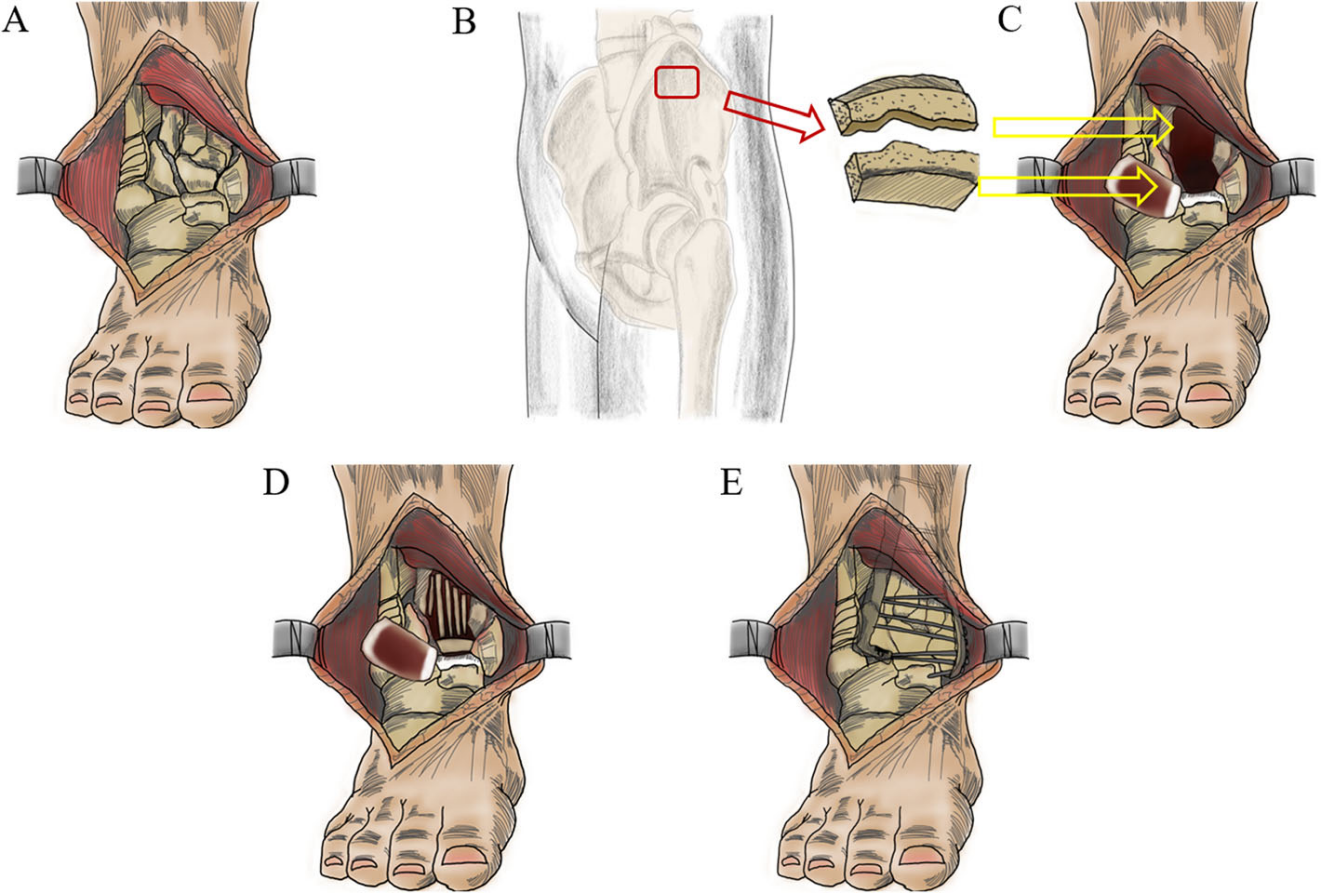
Schematic illustration of the surgical procedure. **a** Rüedi-Allgöwer III or AO/OTA type C3 pilon fractures are severely comminuted fractures with impaction of the distal tibia. **b** A large autologous ilium with periosteum is harvested from the iliac crest. **c** A clear view of the entire plafond and talus is obtained after distraction of the ankle joint with the tensor and removing the fracture fragment. **d** The concave side of ilium with periosteum is implanted directly into the residual subchondral bone using a k-wire, guided by the articular surface of the talus as a template. Cancellous bone from the graft is used to fill the bone defect following reduction of the articular segment. **e** The reconstructed tibiotalar joint and plafond are fixed by anatomically locked plates

This study was performed under approval by the ethics committee of Shanxi Medical University Second Hospital (2018LL036). Twenty-five patients were initially entered into this study, but only twenty were available for evaluation at the final follow-up. Characteristics of the twenty patients included in this study are presented in Table 1. Between March 2015 and September 2018, twenty patients (15 male and 5 female) with a mean age of 45.2 years (range: 19–68) underwent tibiotalar joint reconstruction using a large autologous ilium with periosteum harvested from the iliac crest (Fig. 1). The inclusion criteria were: Rüedi-Allgöwer III or AO/OTA type C3 pilon fractures, Tscherne/Oestern FxCO-I closed fractures and FxOI open fractures [12, 13], no episodes of compartment syndrome, and no exudation or skin shrinkage. The exclusion criteria were: Rüedi-Allgöwer I, II or AO/OTA type C1, C2 pilon fractures, Tscherne/Oestern FxCOII-III closed fractures and FxOII-IV open fractures, and patients lost to follow-up.

**Table 1:** Characteristics of the 20 patients included in this study

Age [mean (range)]	45.2 (19–68)
Sex [number (percentage)]	
Female	5 (25%)
Male	15 (75%)
Mechanism of injury [number]	
Motor vehicle crash	18
Fall	2
Tscherne/Oestern classification [number]	
FxCO closed fracture	5
FxCI closed fracture	13
FxOI open fracture	2
Follow-up time in months [mean (range)]	18.3 (6–36)
Surgical approach [number]	
Anteromedial with back-lateral	5
Anterolateral with back-medial	15
Operation time in minutes [mean (range)]	120 (100–150)
Blood loss in mL [mean (range)]	200 (50–300)

**Fig. 2:**
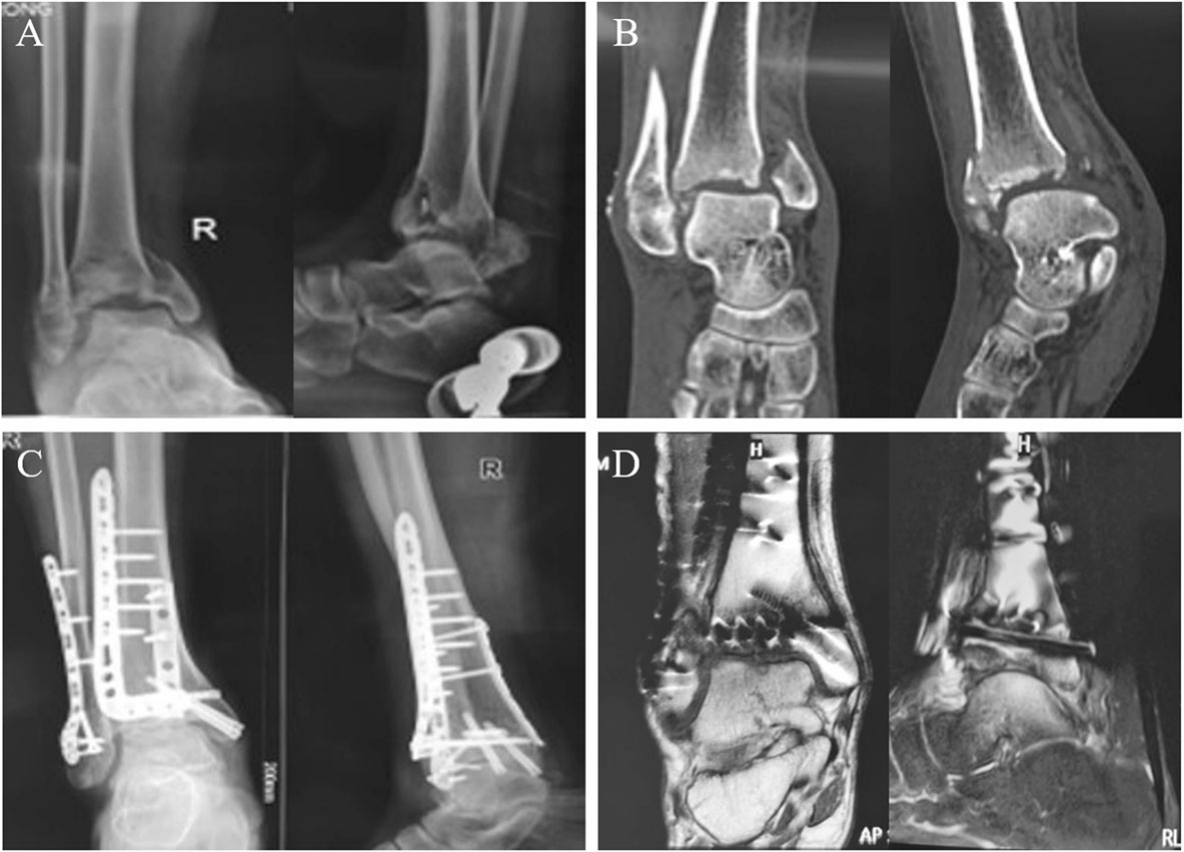
Healing progress of a typical case following reconstruction surgery. **a**, **b** Pre-operative radiograph and CT images of a 19 year old male patient. **c** Post-operative radiograph of the patient at 18 months follow-up. **d**s MRI images showing the integrity and surface of the reconstructed tibiotalar joint

Pre-operative radiographs and 3D reconstructed computed tomography (CT) images were obtained to assess the severity of joint comminution, impaction, and displacement of fracture fragments (Fig. 2a, b). The visual analog scale (VAS) during daily activities and American Orthopaedic Foot & Ankle Society (AOFAS) [14] scores were recorded prior to surgery. These served as baseline data for comparison during follow-ups.

### Surgical technique

Doppler ultrasonography was used to screen for deep vein thrombosis prior to commencing surgery. Surgery was performed under general anaesthesia or combined spinal-epidural anaesthesia in the float position under tourniquet control by a senior surgeon. The fractures were carefully protected and collected, and constructed stepwise according to procedures published by Rüedi and Allgöwer [15]. In 5 patients, ORIF was performed using an anteromedial combined with back-lateral surgical approach [16], while in the other 15 patients an anterolateral combined with back-medial approach was used [17]. Alternative and supplemental approaches were used as required depending on the fracture pattern and soft tissue situation.

A large full-thickness autologous ilium (5 cm × 5 cm) was harvested from the patient’s iliac crest, consisting of cancellous bone with periosteal lamellae on each one side (Fig. 1b), using Wolfe-kawamoto’s technique [18]. The distance between skin bridges was maintained at larger than 7 cm. A clear view of the entire plafond and talus was obtained after distraction of the ankle joint with the tensor and removing the fracture fragment (Fig. 1c). The technique we describe here was to preserve the residual articular cartilage as much as possible, which would then be supported by the iliac crest bone graft. The concave side of ilium with periosteum (about 1 cm thickness) was implanted directly into the residual subchondral bone using a k-wire, with the articular surface of the talus as template. Cancellous bone from the graft was used to fill the bone defect for metaphyseal impaction of bone following reduction of the articular segment (Fig. 1d). In some cases where the cartilage is combined with severe comminuted fracture and cannot be distinctly recognized, the periosteal surface is used to articulate with the surface of the talus.

Fixation of the medial column was performed fundamentally with temporary k-wires, and small fragment screws were applied to give stability to the fracture site. The reconstructed tibiotalar joint and plafond were fixed by anatomically locked pates, which provided initial fixation and the ability to perform soft exercise (Fig. 1e). Donati vertical mattress sutures were used to close the wound [3]. Post-operative radiographs were taken on the next day of surgery and at the latest follow-up.

### Post-operative management

All patients remained in splint or cast immobilisation until the wounds have healed and sutures could be removed. All patients commenced ankle motion exercises the day after operation or as soon as possible. Non-weight bearing movements were introduced thereafter, with progressively increasing amounts of weight bearing (10–15 kg) and range of motion. Patients resumed full weight bearing upon clinician recommendation based on fracture pattern and radiographic findings at follow-up. Prophylaxis for deep vein thrombosis was provided with the use of sequential compression garments and/or low molecular weight heparin throughout the time that the patient was hospitalised and during bed rest, until a maximum period of 14 days.

All patients were required to return for follow-up at 1, 3, 6 and 12 months after surgery, and each year thereafter. At follow-up, the progress of wound healing and any complications were recorded, and outcomes were evaluated using the VAS and AOFAS scores [14], the range of motion of the ankle joint, and radiographs which were used to assess the quality of fracture reduction, stability of fixation, and fracture alignment and union according to Burwell and Charnley criteria [19] (Fig. 2c). Three patients were asked to undergo MRI examination to show the integrity of the distal tibia (Fig. 2d). Implant removal was performed at 1–2 years post-operation, after the bone remodelling process was complete.

## Results

Twenty patients who attended the final follow-up were included in this study. The mean operation time for the surgery was 120 min (range: 100–150), and the average wound recovery time was 16 days (range: 12–21). Two patients developed redness and swelling at the wound site, but recovered after local care and dressing changes. No patients displayed deep surgical site infection, donor site complications, non-union or extraction morbidity from the iliac donor site during follow-up. The mean follow-up duration was 18.3 months (range: 6–36), at which bone union was achieved in all patients. The mean VAS score decreased from 7.7 pre-operatively to 2.8 post-operatively, while the mean AOFAS score improved from 12 to 86. According to the Burwell and Charnley radiological criteria, 15 patients had an anatomic reduction, 4 had good reduction, and 1 had poor reduction. The healing progress of a typical case after surgery is shown in Fig. 2. Clinical outcomes of patients at the final follow-up are summarised in Table 2.

**Table 2:** Clinical outcomes at final follow-up of 6 months or later post-operation

Average healing time in days [mean (range)]	16 (12–21)
Soft tissue complications [number]	
Redness and swelling	2
Superficial wound infection	0
Deep wound infection	0
Extraction morbidity from Iliac Donor Site	0
Bone union time in months [mean (range)]	18.3 (3–36)
Average VAS score	
Pre-operative	7.7 (6.9–9)
Post-operative	2.8 (0–4)
Avera ge score on AOFAS	
Pre-operative	12
Post-operative	86
Burwell and Charnley results [number]	
Anatomic reduction	15
Good reduction	4
Poor reduction	1

## Discussion

Pilon fractures frequently lead to post-traumatic ankle osteoarthritis, with an incidence of up to 33–50% [20]. For treating Rüedi-Allgöwer III or AO/OTA type C3 pilon fractures, a consensus has not been reached in the literature regarding the optimal fixation method. The primary cause is unsatisfactory reduction of tibial plafond fractures [21], which results in elevated contract stress at the tibiotalar joint [22]. In this study, we report one experimental technique for tibiotalar joint reconstruction using large autologous ilium with periosteum that can be applied for the treatment of Rüedi-Allgöwer III or AO/OTA type C3 pilon fractures. The technique described allows for direct visualization and optimal reduction of both the articular surface and the metadiaphyseal component of the injury. Although the use of external fixation can be accomplished rapidly and limit additional soft tissue injury, this may also increase the likelihood of tibiotalar or subtalar joint stiffness [23]. Since accurate reduction of a displaced articular fracture is considered to have critical importance in minimizing the risk of developing post-traumatic osteoarthritis, we recommend that ORIF be performed after evaluating the soft tissue condition. This technique of combining an autologous ilium having periosteum with ORIF achieved satisfactory healing outcomes at greater than 6 months follow-up in all included patients, with preservation of joint function in the injured ankle and restoration of the patients’ ability to perform daily activities. In the long-term, better reconstruction may reduce the risk of developing degenerative changes in the tibiotalar joint, thus delay joint fusion or articular arthroplasty in the future.

Fresh articular allografts are frequently used for knee reconstruction following trauma, such as for fractures of the tibial plateau [24]. However, allografts are typically only indicated for young patients with significant articular defects, and their use to treat large tibiotalar defects in Rüedi-Allgöwer III or AO/OTA type C3 pilon fractures are seldomly reported. In this study, we reconstructed the tibiotalar joint using autologous ilium having periosteum harvested from the iliac crest, which are the gold standard of repair for defects with large amounts of bone loss. The success of this procedure partly depends on the time course of rehabilitation, where patients are advised to commence ankle motion exercises as soon as possible after operation. The mechanism for satisfactory articular reconstruction using concave side of ilium with periosteum is likely related to the influence of mechanical stress on joint tissue repair, which helps with the gradual transformation of granulation tissue at the defect site into bone tissue, fibrocartilage, and even hyaline cartilage. However, the characterization at 18 months in this study does not give definitive evidence of the repair tissue composition, and longer-term follow-up with noting of possible complications such as arthritic changes are necessary [24, 25]. Nevertheless, the restoration of ankle movement may introduce physiological loading that is beneficial to the healing and grinding of the articular surface, and reduce the risk of developing post-traumatic osteoarthritis [26].

Although we have demonstrated satisfactory outcomes in this study, the use of the described technique for tibiotalar joint reconstruction should be considered with caution. It has been reported that complete reduction in pilon fractures can lead to complete failure, such as non-union, avascular necrosis and osteomyelitis [27]. Some precautions that should be taken when employing this technique include: (1) the articular surface of the talus should be used as a template for reconstructing the joint relationship; (2) the residual inner and outer surrounding fractures should be restored to provide support to the bone autograft; and (3) a small bone hook can be applied to restore the vertical displacement of the joint bone fragment and recover the flatness of the articular surface. In this study, we found that a 7 cm skin bridge was required even with proper soft tissue management and appropriate timing of surgical intervention. However, other studies have suggested that the pattern of injury, rather than skin bridge dimension, should dictate the selection of surgical approach, and that a skin bridge of less than 7 cm can be used [28]. We also found that the anterolateral combined with back-medial approach was suitable for most patients and achieved good outcomes, which was in agreement with observations in other studies [17].

There were some limitations in our study. First, there was variation in the length of follow-up among the included patients, which ranged from 3 to 36 months. Combined with the small sample size, we were not able to obtain sufficient information on long-term joint status or draw definitive conclusions on the safety and efficacy of the reconstructive technique, but to assess the feasibility of our technique as a pilot study. No complications in 20 cases would give an upper 95% confidence interval of approximately 15% of not having a complication, and one poor reduction an upper 95% confidence interval of approximately 25%. The patients were also not assessed for the development or severity of post-traumatic osteoarthritis at follow-up evaluations. Second, the clinical outcomes were mainly based on subjective patient experience, as it was difficult to apply standardised objective measurements of ankle function and then to anticipate the probability of occurrence of ankle arthritis. Third, this study only included a small and selected cohort of patients with high-energy pilon fracture. The safety and efficacy of the described technique needs to be verified in a larger sample of patients across different research centers, and over consistent long-term time frames to generate more high-level evidence (by using random control trail studies) to inform its usefulness in the reconstruction of high-energy pilon fractures.

## Conclusion

The use of a large autologous ilium with periosteum together with ORIF for tibiotalar joint reconstruction is an experimental technique for the treatment of Rüedi-Allgöwer III or AO/OTA type C3 pilon fractures, and may reduce post-operative complications in short-term follow-up.

## Data Availability

The datasets used and analyzed during the current study are available from the corresponding author on reasonable request.

## Abbreviations

ORIF: Open reduction and internal fixation
VAS: Visual analog scale
AOFAS: American Orthopaedic Foot & Ankle Society
MIPO: Minimally invasive plate osteosynthesis
CT: Computed tomography
